# Designing clinical trials for assessing the effects of cognitive training and physical activity interventions on cognitive outcomes: The Seniors Health and Activity Research Program Pilot (SHARP-P) Study, a randomized controlled trial

**DOI:** 10.1186/1471-2318-11-27

**Published:** 2011-05-26

**Authors:** Claudine Legault, Janine M Jennings, Jeffrey A Katula, Dale Dagenbach, Sarah A Gaussoin, Kaycee M Sink, Stephen R Rapp, W Jack Rejeski, Sally A Shumaker, Mark A Espeland

**Affiliations:** 1 Division of Public Health Sciences, Department of Biostatistical Sciences - Wachovia 21st floor, Medical Center Boulevard, Winston-Salem, North Carolina, 27157, USA; 2 Department of Psychology, Wake Forest University, Winston-Salem, North Carolina, 27101, USA; 3 Department of Health and Exercise Sciences, Wake Forest University, Winston-Salem, North Carolina, 27101, USA; 4 Sticht Center on Aging and Wake Forest University School of Medicine, Winston-Salem, North Carolina, 27157, USA; 5 Department of Psychiatry and Behavioral Medicine, Wake Forest University School of Medicine, Winston-Salem, North Carolina, 27157, USA; 6 Division of Public Health Sciences, Department of Social Sciences and Health Policy, Medical Center Boulevard, Winston-Salem, North Carolina, 27157, USA

## Abstract

**Background:**

The efficacy of non-pharmacological intervention approaches such as physical activity, strength, and cognitive training for improving brain health has not been established. Before definitive trials are mounted, important design questions on participation/adherence, training and interventions effects must be answered to more fully inform a full-scale trial.

**Methods:**

SHARP-P was a single-blinded randomized controlled pilot trial of a 4-month physical activity training intervention (PA) and/or cognitive training intervention (CT) in a 2 × 2 factorial design with a health education control condition in 73 community-dwelling persons, aged 70-85 years, who were at risk for cognitive decline but did not have mild cognitive impairment.

**Results:**

Intervention attendance rates were higher in the CT and PACT groups: CT: 96%, PA: 76%, PACT: 90% (p=0.004), the interventions produced marked changes in cognitive and physical performance measures (p≤0.05), and retention rates exceeded 90%. There were no statistically significant differences in 4-month changes in composite scores of cognitive, executive, and episodic memory function among arms. Four-month improvements in the composite measure increased with age among participants assigned to physical activity training but decreased with age for other participants (intervention*age interaction p = 0.01). Depending on the choice of outcome, two-armed full-scale trials may require fewer than 1,000 participants (continuous outcome) or 2,000 participants (categorical outcome).

**Conclusions:**

Good levels of participation, adherence, and retention appear to be achievable for participants through age 85 years. Care should be taken to ensure that an attention control condition does not attenuate intervention effects. Depending on the choice of outcome measures, the necessary sample sizes to conduct four-year trials appear to be feasible.

**Trial Registration:**

Clinicaltrials.gov Identifier: NCT00688155

## Background

There is growing interest in non-pharmacological interventions for preventing, reducing, or postponing cognitive decline in late life [1-3]. The efficacy of approaches such as physical activity, strength and cognitive training for improving brain health has not been established [4]; however, results from small, short-term trials are encouraging and support the larger, more definitive trials necessary to establish efficacy and prevention guidelines [5-16].

Before definitive trials are mounted, it is important to conduct pilot studies that address important practical concerns. These include assessing whether recruitment approaches are successful and quantifying their expected yields; examining whether high levels of adherence can be maintained when interventions are multi-factorial; obtaining experience with an appropriate control condition, to evaluate whether it promotes retention and how well it serves as a comparator; examining whether the retention, adherence, and relative effectiveness of training-based interventions vary depending on participant's age; and obtaining the information necessary to project the required sample size for potential outcomes. The Seniors Health and Activity Research Program Pilot trial (SHARP-P) was designed to serve these purposes for trials assessing cognitive and physical activity training. We report its primary results.

## Methods

SHARP-P was a single-blinded pilot randomized controlled trial that involved the delivery of a cognitive training intervention and/or a physical training intervention in a 2 × 2 factorial design. Its design was approved by the Institutional Review Board of Wake Forest University and all participants signed an informed consent. It targeted the enrollment of 80 community-dwelling persons, aged 70-85 years, who were at risk for cognitive decline but who did not have mild cognitive impairment [17] as defined by the inclusion/exclusion criteria related to cognition (Table 1). Other criteria identified individuals who were appropriate candidates for physical activity and cognitive training and who appeared likely to adhere to interventions and data collection protocols [18].

**Table 1:** Exclusion criteria for the Seniors Health and Activity Research Program Pilot Trial.

Exclusion Criteria Related to Physical Activity
Telephone Screening Visits
◦ Severe rheumatologic or orthopedic diseases
◦ Severe pulmonary disease
◦ Actively participating in a formal exercise program within the past month (defined as >30 min/week)
◦ Severe cardiac disease, including NYHA Class III or IV congestive heart failure, clinically significant aortic stenosis, history of cardiac arrest which required resuscitation, use of a cardiac defibrillator, or uncontrolled angina
◦ Other significant co-morbid disease that would impair ability to participate in the exercise-based intervention
◦ Receiving physical therapy for gait, balance, or other lower extremity training
◦ Myocardial infarction, Coronary Artery Bypass Graft, or valve replacement within past six months
◦ Serious conduction disorder (e.g. 3^rd ^degree heart block), uncontrolled arrhythmia
◦ Pulmonary embolism or deep venous thrombosis within past 6 months
◦ Hip fracture, hip or knee replacement, or spinal surgery within past 4 months
◦ Severe hypertension
Clinic Visits
◦ None
**Exclusion Criteria Related to Cognition**
**Telephone Screening Visits**
◦ Neurologic disease, e.g. Alzheimer's disease (or other types of dementia), stroke that required hospitalization, Parkinson's, multiple sclerosis, Amyotrophic Lateral Sclerosis, or Mild Cognitive Impairment
◦ Telephone Interview for Cognitive Status (TICS) ≤ 31
◦ Current use of cognitive enhancing prescription or investigational medications
◦ History of participation in a cognitive training program in the last two years
**Clinic Visits**
◦ 3MSE score <88 (<80 for ≤8 years education)
◦ Scores ≥2 standard deviations below normal on memory or non-memory domain tests (speed of processing and verbal fluency)
◦ Other significant factors that may affect the ability for cognitive training, including a history of head trauma resulting in a loss of consciousness, current use of benzodiazepines, hypnotic or anticholinergic agents
◦ Stroke within past 4 months
◦ Baseline Geriatric Depression Scale score ≥8

**Exclusion Criteria Related to Trial Design or Adherence**
**Telephone Screening Visits**
◦ Age <70 or >85 years
◦ Unwillingness to be randomized to any of the four intervention conditions
◦ Failure to provide the name of a personal physician
◦ Living in a nursing home
◦ Terminal illness with life expectancy less than 8 months
◦ Unable to communicate because of severe hearing loss or speech disorder
◦ Severe visual impairment
◦ Excessive alcohol use (>14 drinks per week)
◦ Member of household is already enrolled
◦ Lives distant from the study site or is planning to move out of the area in the next year or leave the area for more than one month during the next year
◦ Other temporary intervening events, such as sick spouse, bereavement, or recent move
◦ Participation in another intervention trial
**Clinic Visits**
◦ Inability to commit to intervention schedule requirements
◦ Failure to provide informed consent

**Figure 1:**
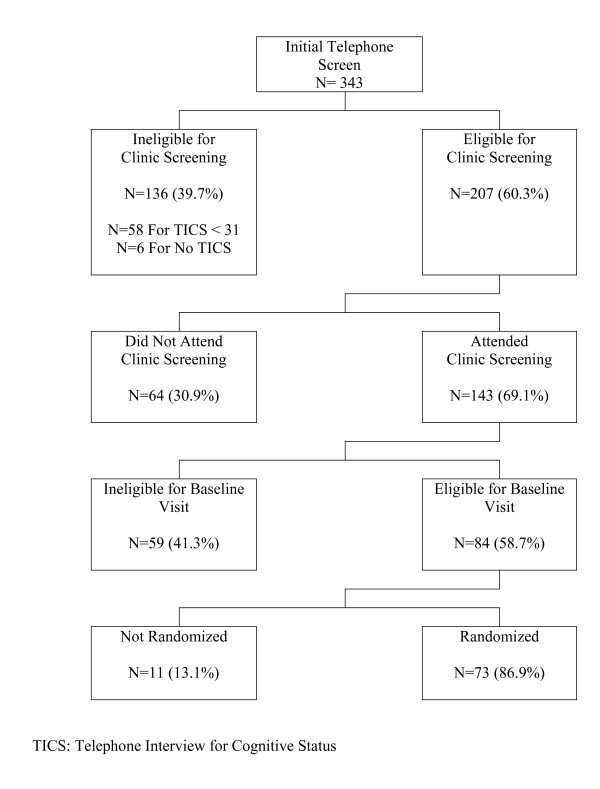
**Participant flow through the trial**.

Enrollment proceeded in four steps. Mailing and presentations were used to identify interested volunteers. An initial screening phone call was used to query regarding major sources of exclusions. During a subsequent clinic screening visit, cognitive testing was used to exclude individuals with marked cognitive deficits. Current medications were reviewed to exclude those using anticholinergic agents, antidepressants, antihypertensives (clonidine or catapres only), anti-Parkinsonian agent, narcotic analgesics, neuroleptics, sedatives/benzodiazepines, and dementia drugs. Volunteers who remained eligible and received clearance from their personal physicians were invited to a final visit for additional data collection. Following this, they were randomly assigned with equal probability among the four experimental conditions. Figure 1 provides a pictorial overview of this process.

### Interventions

The Cognitive Training (CT) intervention was developed to improve consciously-controlled memory processing or recollection of episodic memory information [19] and produce changes in performance that transfer to executive function, such as working memory, planning, and memory monitoring; long term item memory; and cognitive processing speed [20]. Sessions were center-based, conducted via computer, carried out with small groups of no more than six individuals, and monitored by skilled trainers. Training consisted of four consecutive 10-12 min sessions per day, administered two times per week for two months, which then tapered to one time per week for two additional months. For each session, participants studied a list of 30 words, followed by a recognition test consisting of the 30 studied words and 30 new words with each new word repeated once, and asked to respond "yes" to study words and "no" to the new items both times they occurred. The second presentation of new items was critical as participants had to consciously recollect the source of a word's presentation (studied or not) or whether they had already responded to a word to correctly respond "no". To improve memory during training, participants started at a level where recollecting the first presentation of a repeated word was relatively easy (i.e., only one intervening item between the 1^st ^and 2^nd ^presentation of a repeated word), and underwent an increase in lag interval length each time they reached a criterion level of accuracy [19,20]. Training gains were assessed by the number of intervening items (i.e., lag interval) at which participants could perform to criterion between the first and last day of training. We chose this intervention as it has been shown to be successful with older adults in enhancing the ability to recollect information across increasing delay intervals [20] and in improving performance on tasks that draw on executive function [19].

The Physical Activity (PA) Training intervention consisted of center-based and home-based sessions aimed primarily on aerobic and flexibility training with a targeted duration of 150 minutes/week. It included two center-based training sessions per week for four months. Its primary focus was walking with the explicit intent of improving cardiovascular fitness. Other forms of endurance activity (e.g., stationary cycling) were used when regular walking was contraindicated for medical or behavioral reasons. Center-based physical activity sessions were approximately 60 minutes and consisted of a 40 minute walking stimulus phase and a 20-minute flexibility training phase. Center-based physical activity was supplemented with additional tailored home-based walking sessions at 1-2 per week during the first month. Participants were encouraged to slowly increase the duration, speed, and frequency of home-based walking sessions as appropriate to their circumstances so as to achieve the 150-min/week goal. The interventionists recorded center-based walking time and the participants recorded home-based walking time on physical activity logs that were returned to the interventionists once a week.

The Combined Intervention (PACT) was designed so that participants received both cognitive and physical activity training on the same day. To avoid the potential impact of physical fatigue on cognitive training, the cognitive treatment was delivered prior to the physical activity treatment.

The Healthy Aging Education control intervention consisted of weekly lectures based on health education and was based on a program developed originally at Stanford [21] and adapted for the Lifestyle Interventions and Independence for Elders pilot trial [22,23]. Topics such as medications, foot care, traveling, and nutrition were covered.

### Cognitive Testing

Six measures of executive functioning were included. The Self-Ordered Pointing Task [24] measured planning, working memory and monitoring. Participants viewed a set of 16 abstract shapes presented in a different random order 16 times. For each of three trials, they were required to choose a shape so that every shape was selected by the 16^th ^trial and no shape was chosen more than once. The 1-Back and 2-Back Tests [25,26] measured working memory. Participants saw individual letters at a 2-second rate on a computer screen and were asked to indicate whether the presented letter was the same as the nth back letter, with n equal to 1 and 2. The Eriksen flanker task [27] measured response inhibition. Participants were presented with an arrow facing either right or left and were asked to press a key indicating its direction. The target displays could be congruent (flanker arrows point in the same direction as the target arrow) or incongruent (the flanker arrows point in the opposite direction). The Task Switching test [28] measured attentional flexibility. Participants were asked to quickly alternate between performing two different tasks. They were shown a letter-number pair (e.g., T 5) inside one box of a 4-box grid. When the stimulus appeared in either of the top two boxes, the participant indicated whether the number was odd or even; when the stimulus appeared in either of the two lower boxes, s/he indicated whether the letter was a vowel or consonant. The Trail Making Test was also used to measure alternating attention and executive function [29]. Participants had a maximum of five minutes to connect 25 numbered circles in ascending order (Part A) and another maximum of five minutes to connect alternatively numbered and alpha-numerically labeled (1-A-2-B,...) circles (Part B). They were scored by the time it took to complete the task, and Part A scores were then subtracted from Part B.

Four measures of episodic memory derived from the Hopkins Verbal Learning Test [30] and the Logical Memory task from the Wechsler Memory Scale-III [31] were also included. The Hopkins Verbal Learning Test required individuals to listen to a list of 12 words and repeat as many as possible. Three trials were administered. After a delay of about 15 minutes, participants were asked to recall the elements. Participants were also presented a yes/no delayed recognition trial consisting of a randomized list that included the 12 target words and 12 non-target words, six of which were drawn from the same semantic categories as the targets. Scores for delayed recall and delayed recognition were calculated. For Logical Memory Part I, participants listened to a short story and immediately recalled as many elements as possible. The individual was then read a second story twice and asked to immediately recall as many elements as possible each time. Logical Memory Part II was administered after a delay of approximately 25 to 35 minutes. Individuals were asked to recall the elements of both stories, again. For both Logical Memory I and II tasks, individuals received a "story unit score" for accuracy of re-telling the story details and a "thematic score" for recalling story themes. A primary score was calculated from a sum of the "story unit scores". A supplemental score was calculated from a sum of the '"thematic scores".

A composite of the ten measures described above was computed by dividing each score's difference from the baseline mean by the baseline standard deviation, averaging the six executive function and four episodic memory z-transformed measures, and norming this average to have standard deviation of one. The primary outcome for SHARP-P was change in this composite from baseline at four months post-randomization. The separate domains of executive function and episodic memory served as secondary outcome measures.

### Other baseline measures

A timed 400-meter walk was used as a measure of physical function. The Geriatric Depression Scale was administered as well as a Modified Mini Mental State Exam (3MSE) [32] to measure global cognitive function. ApoE genotyping was performed using the MassARRAY SNP genotyping system (Sequenom, Inc., San Diego, CA). Genotypes were determined by mass spectroscopy (MALDI-TOF). The data were analyzed using SpectroTYPER software (Sequenom). Controls and blanks were included for quality and error checking.

### Statistical Methods

All analyses were conducted according to intention-to-treat principles. Differences among participants assigned to the four intervention conditions were contrasted using analyses of variance and Fisher's exact tests. Cognitive function data were converted to z-scores and analyses of variance/covariance were used to assess differences in mean changes among participants grouped by intervention assignment following intention-to-treat principles.

The trial protocol pre-specified subgroup comparisons based on age, education, and presence of the apoE allele, which were assessed using interaction terms. The sample size targeted for SHARP-P (80 participants; 5% lost follow-up rate) was chosen to provide >80% power to detect an effect size of 20% on the marginal means.

## Results

### Participants

SHARP-P screened 343 participants to enroll 73 (21%) participants, with randomizations occurring from September, 2008 through July, 2009 (Figure 1). Major sources of ineligibility were self-reported exercise levels of ≥30 minutes/week (9%), history of severe chest pain (4%), congestive heart failure (4%), and hospitalization for stroke (4%). Table 2 describes the cohort at baseline by intervention assignment. Overall, the cohort had mean age 76.4 years, 51% were women, and 75% had some education post-high school. The mean 3MSE score was 94.8 and the mean 400-meter walk time was 342 seconds. There were no marked imbalances among randomly assigned groups with respect to any of the cognitive function measures at baseline. There was only one serious adverse event in which a participant had a stroke following randomization, before receiving any training, and dropped out.

**Table 2:** Characteristics of SHARP-P volunteers, by intervention assignment: mean (standard deviation) or percent.

Characteristic	Healthy Aging N = 18	Cognitive Training N = 18	Physical Activity Training N = 18	Combined Intervention N = 19	p-value^1^
Age, years	75.4 (4.8)	76.0 (5.2)	77.5 (4.8)	76.9 (4.0)	0.57
Sex					
Female	7 (38%)	8 (44%)	10 (56%)	12 (63%)	0.45
Male	11 (61%)	10 (56%)	8 (44%)	7 (37%)	
Education					
HS or less	5 (28%)	4 (22%)	3 (17%)	6 (32%)	0.74
> HS	13 (72%)	14 (78%)	15 (83%)	13 (68%)	
Race/ethnicity					
African-American	1 (6%)	1 (6%)	1 (6%)	4 (21%)	0.45
Caucasian	17 (94%)	17 (94%)	17 (94%)	15 (79%)	
3MSE	94.3 (2.4)	95.6 (3.4)	94.6 (3.9)	94.6 (4.3)	0.19
400 m walk, secs	331 (66)	331 (50)	360 (48)	347 (56)	0.36
ApoE allele^2^					
Absent	9 (75%)	10 (83%)	12 (86%)	10 (62%)	0.44
Present	3 (25%)	2 (17%)	2 (14%)	6 (38%)	

^1^Analyses of variance for continuous measures; Pearson's chi-square or Fisher's exact tests for categorical variables

^2^Missing for 19 participants

^3^3MSE: Modified Mini Mental State Exam

### Training

**Figure 2:**
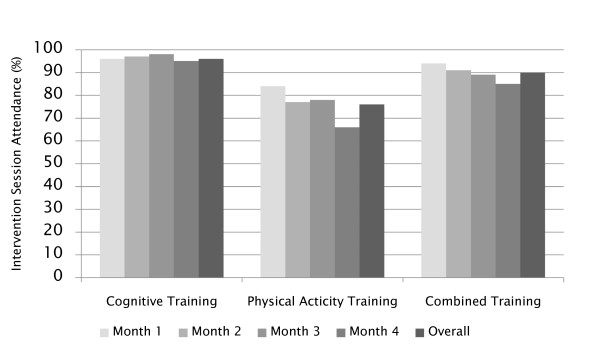
**Intervention attendance rates (of expected) by month and overall**. There was a significant overall trend for attendance rates to decline over time (p = 0.002).

For cognitive training, 24 sessions were planned for each participant (8 per month during Months 1-2 and 4 per month during Months 3-4). For physical activity training, 32 sessions were planned (8 visits per month across Months 1-4). For the combination training, 56 sessions were planned (24 cognitive training and 32 physical activity training sessions, as above). Figure 2 portrays the attendance rates for the three interventions. Overall attendance rates were higher in the CT and PACT groups: CT 96%, PA 76% and PACT 90% (p = 0.004). The highest rates were observed for the cognitive intervention and rates tended to decline over time (p = 0.002). Overall attendance at clinic sessions was uncorrelated with age: r = -0.18 (p = 0.20).

#### Cognitive Training

Thirty-three participants assigned to cognitive training attended at least one session and 91% attended all 24 sessions. Improvement in the cognitive training task was assessed by the maximum lag interval between initial presentation of a new study item and its subsequent reappearance in the test list with the maximum possible lag interval of 52 intervening items. At baseline, the average maximum lag (standard error) was 3.0 (0.43) in the CT group and 2.1 (0.39) items in the PACT group (p = 0.11). Task performance increased significantly among participants assigned to CT and PACT (p < 0.0001). The mean difference between the maximum lag of the last session and the maximum lag of the first session was 43.9 (3.3) items for the CT group compared to 29.2 (4.5) items for the PACT group (p = 0.02). Neither age nor ApoE e4 were associated with differences in lags: r = 0.05 (p = 0.79) and r = -0.15 (p = 0.46), respectively. Participants with education higher than high school had significantly higher average difference in lags than those with less education: 40.4 (4.4) versus 24.0 (5.6) items, p = 0.02.

#### Physical Activity

The physical activity intervention had a goal of inducing 150 minutes of physical activity per week to be achieved via both center-based and home-based sessions. Two participants in the PA group were excluded as they did not return for their 4-month visit and one participant in each of the PA and PACT group were also excluded as they did not attend any of the center-based sessions. For all other participants, zero minutes were assigned for a missed session. The overall mean (standard error) number of minutes per week across the 4 months of follow-up was 57.2 (5.0) for the PA group and 57.3 (3.6) for the PACT intervention (p = 0.99). There was no significant association between minutes of physical activity and age (r = -0.09, p = 0.61) or ApoE e4 (r = -0.05, p = 0.79). High school education did not increase the average number of minutes either: 59.7 (6.9) for those with a high school degree and 56.4 (3.9) for those without (p = 0.68). Participants in the PACT group tended to exercise at home longer on average than those in the PA group: 101.1 (13.1) minutes per week in the combined group and 66.0 (12.6) minutes per week in the PA group (p = 0.06). Combining center-based and home-based physical activity, participants in the PA condition completed an average of 123.2 (16.8) minutes per week of total physical activity and participants in the PACT condition completed an average of 135.9 (15.3) minutes per week (p = 0.58). Participants assigned to physical activity decreased the 400-meter walk times by a mean (standard error) of 16.9 (5.31) seconds (p = 0.05) and there was no difference between PA and PACT (p = 0.74). Walk times of participants assigned to HAE (p = 0.18) or CT (p = 0.80) did not improve.

#### Cognitive Outcomes

Table 3 lists means for the cognitive test measures during follow-up. The top panel includes tests that contributed to the overall executive function score; these were measured at both two and four months post-randomization, however only the four month data are included in our analyses for consistency with the measures that contributed to the overall episodic memory score in the bottom panel (which were only measured at four months).

**Table 3:** Scores on tests of cognitive function, by intervention assignment and time: mean (standard deviation)

A) Measures of Executive Function					
**Outcome**	**Month**	**Healthy Aging** **N = 17**	**Cognitive Training** **N = 16**	**Physical Activity Training** **N = 16**	**Combined Intervention** **N = 18**

Self-Ordered Pointing Task (proportion correct)	0	0.71 (0.05)	0.74 (0.06)	0.73 (0.09)	0.71 (0.10)
	4	0.74 (0.06)	0.77 (0.06)	0.76 (0.07)	0.75 (0.08)
					
1-Back (proportion Hits - False Alarms)	0	0.87 (0.13)	0.88 (0.08)	0.89 (0.14)	0.84 (0.12)
	4	0.93 (0.06)	1.00 (0.25)	0.92 (0.08)	0.89 (0.14)
					
2-Back (proportion Hits - False Alarms)	0	0.48 (0.22)	0.60 (0.17)	0.55 (0.21)	0.57 (0.19)
	4	0.61 (0.19)	0.62 (0.28)	0.57 (0.18)	0.62 (0.18)
					
Flanker Task (Incongruent - Congruent RTs)	0	31.7 (54.3)	38.9 (22.8)	31.8 (26.0)	63.3 (68.1)
	4	40.0 (39.8)	20.1 (30.2)	25.2 (23.9)	36.6 (29.8)
					
Task Switching (Switch - Non-switch RTs)	0	893.7 (505.0)	720.3 (388.9)	888.4 (738.4)	874.9 (640.9)
	4	739.5 (403.0)	672.3 (353.1)	814.3 (689.9)	743.3 (554.1)
					
Trails B Time - Trails A Time	0	74.8 (54.4)	54.4 (30.0)	43.1 (33.1)	63.4 (54.5)
	4	51.5 (22.3)	52.4 (30.0)	46.1 (69.0)	64.4 (51.2)
					
**B) Measures of Episodic Memory**					

**Outcome**	**Month**	**Healthy Aging** **N = 17**	**Cognitive Training** **N = 16**	**Physical Activity Training** **N = 16**	**Combined Intervention** **N = 18**

HVLT - Immediate recall	0	22.4 (1.0)	22.2 (1.4)	23.5 (4.2)	22.0 (1.7)
	4	21.8 (1.7)	22.2 (2.2)	22.7 (0.9)	22.4 (2.2)

HVLT - Delayed recall	0	7.4 (1.3)	8.5 (2.1)	7.8 (2.2)	7.5 (2.7)
	4	8.5 (2.8)	9.4 (2.2)	9.4 (2.2)	8.2 (2.5)

LM1 - Supplemental Score - 1st Recall	0	23.5 (6.6)	23.5 (6.5)	23.5 (4.2)	23.4 (4.3)
	4	26.6 (6.3)	26.5 (6.8)	28.3 (5.9)	25.8 (5.3)

LM2 - Recall Total Score	0	21.4 (5.6)	24.1 (7.2)	23.5 (6.1)	21.9 (6.2)
	4	26.0 (5.9)	25.8 (6.8)	28.1 (6.9)	25.8 (6.6)

HVLT: Hopkins Verbal Learning test

LM: Logical Memory

Table 4 summarizes 4-month changes in the composite score and the executive function and episodic memory components. Included are 95% confidence intervals for mean changes, the majority of which exclude 0 and signal general improvements in test scores from baseline across all intervention conditions. None of the marginal comparisons, i.e. physical activity training versus no physical activity training and cognitive training versus no cognitive training, approached statistical significance. Similarly, none of the tests for interactions in the factorial design approached statistical significance.

**Table 4:** Mean change at Month 4 (follow-up - baseline) in standard deviations units.

Outcome	Healthy Aging N = 17	Cognitive Training N = 16	Physical Activity Training N = 16	Combined Intervention N = 19	p-values		
	
	Mean (SE) [95% CI]	Mean (SE) [95% CI]	Mean (SE) [95% CI]	Mean (SE) [95% CI]	CT vs No CT	PA vs No PA	Interaction
Composite	0.65 (0.18)	0.62 (0.18)	0.71 (0.18)	0.71 (0.17)			
	[0.30, 0.99]	[0.26, 0.97]	[0.35, 1.06]	[0.39, 0.97]	0.95	0.66	0.91
							
Executive Function	0.58 (0.19)	0.58 (0.19)	0.32 (0.19)	0.60 (0.18)			
	[-0.00, 0.76]	[0.20, 0.96]	[-0.06, 0.70]	[0.24, 0.94]	0.48	0.55	0.47
							
Episodic Memory	0.47 (0.21)	0.42 (0.21)	0.84 (0.21)	0.56 (0.20)			
	[0.06,0.87]	[0.05, 0.89]	[0.42, 1.26]	[0.17, 0.94]	0.42	0.23	0.58

**Figure 3:**
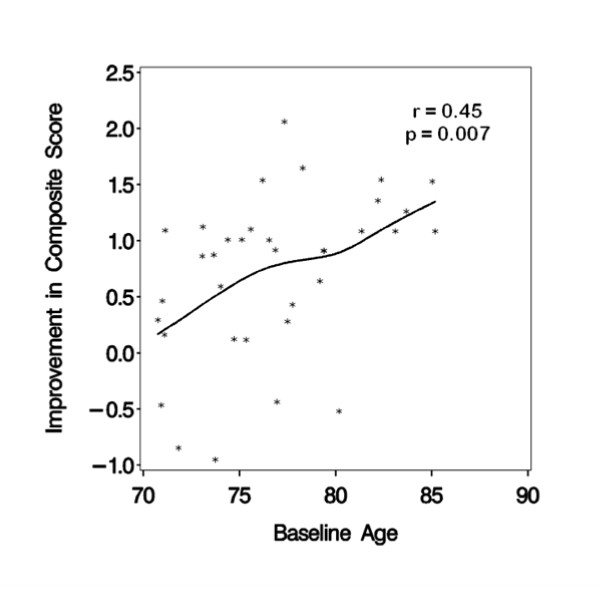
**Relationship between improvement in cognitive function and age with the physical activity intervention**.

**Figure 4:**
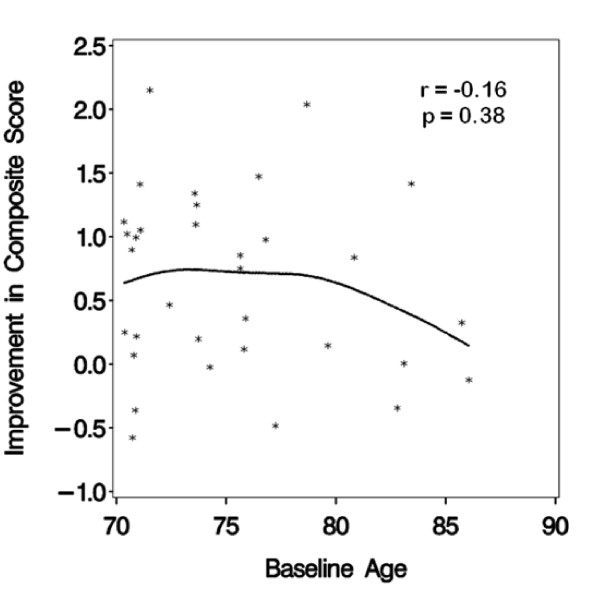
**Relationship between improvement in cognitive function and age with no physical activity intervention**.

An aim specified in the SHARP-P protocol was to examine whether the interventions appeared to perform uniformly well across the age range of the cohort. With age as a continuous variable, formal tests for interactions between age and the cognitive and physical activity intervention effects on the composite, executive function, and episodic memory outcomes were assessed. There was little evidence that the relative effects associated with the cognitive training intervention differed by age: tests of interactions yielded p-values of p = 0.93 (composite), p = 0.56 (executive function), and p = 0.48 (episodic memory). However, there was some evidence that the relative effects of the physical activity intervention were age-dependent: tests of interactions yielded p = 0.01 (composite), p = 0.048 (executive function), and p = 0.11 (episodic memory). Figures 3 and 4 portray the associations, using a scatterplot that includes a curve from cubic spline regression. Among participants assigned to physical activity training, measured improvements in the composite score increased across the age range; among those not assigned to physical activity training, improvements decreased with age.

The trial protocol also pre-specified comparisons of relative intervention effects for subgroups based on education and the presence of the Apo-E allele. Analyses similar to those conducted for age did not reveal any differences in the associations between ApoE e4 and the composite, executive and episodic outcomes between the different interventions (p = 0.70, 0.95 and 0.44 respectively) nor did the analyses show group differences for the association of education with the memory outcomes (p = 0.35, 0.56 and 0.57 respectively). The 4-month change in composite score was 0.62 (0.12), 0.79 (0.24) and 1.0 (0.51) for ApoE e4 equal to 0, 1 or 2, respectively (p = 0.65). It was 0.62 (0.10) for those with more than high school education and 0.85 (0.18) for those below high school (p = 0.26).

#### Sample Size Projections

The pilot trial was designed to provide benchmarks from which to project the sample size required for a full scale trial. These include longitudinal correlations of measures, how cross-sectional variance may change with time, and anticipated rates of follow-up. For the composite outcome of SHARP-P, the longitudinal correlation between baseline and four months was r = 0.89. Variances of cognitive measures were expected to be compressed at baseline due to eligibility criteria. The composite measure variance increased by 6% between baseline and four months. The 4-month missing data rate for the composite outcome was 7%. These data can be used to project required sample sizes for a full scale trial, as follows. We simulated 20,000 sequences of (normally-distributed) annual measures, assuming a mean intervention effect that radiated linearly from baseline to be 0.20 standard deviations at four years, with the assumption that the longitudinal correlations were r = 0.80 (assumed to be lower than in SHARP-P due to the longer time spans), the cross-sectional variance increased by 15% post-randomization (assumed to be larger than in SHARP-P, for the same reason), and the lost follow-up rate accumulated at 8%/year. From these, we projected the sample size required for 90% statistical power to detect differences in a mean relative intervention across follow-up in a two-armed trial (two-sided, unadjusted significance level of 0.05) from a repeated measures model. This required the randomization of N = 290 per group. In a parallel fashion, we projected the sample size required for 90% power for a categorical outcome: measured decline ≥1.0 standard deviation from baseline in the composite outcome. This required the randomization of N = 896 per group to detect a 50% reduction in the rate of this outcome with 4 years of follow-up.

## Discussion

In preparation for a subsequent full scale research study, we discuss further the recruitment, adherence and retention, the choice of a control condition, the effects of cognitive and physical activity intervention on cognition and their consistency with age, as well as predictions of future sample sizes based on the pilot data.

### Recruitment for trials of training-based interventions

SHARP-P recruitment was based on mailing lists and presentations at health education meetings. SHARP-P randomized 73 out of 343 participants aged 70-85 years who were screened on the telephone for a yield of 21.3%. While this yield exceeds that of some other pilot trials [11,23], others who have used a greater breadth of approaches such as print advertisements and television features [10] or who have targeted institutionalized cohorts [12] have achieved higher yields (e.g., 45%-60%).

### Adherence and Retention

Attendance rates were higher in the cognitive training group and the combined group than in the physical activity group, with the highest rates in the cognitive training group. However, this difference may be due to the availability of make-up sessions in the cognitive training condition. Participants were not provided the opportunity to make up missed physical activity visits. Attendance declined only slightly over time except in the physical activity alone arm where the decline tended to be more pronounced. SHARP-P physical activity attendance is similar to that observed in LIFE-P using a tapered contact schedule: 76% during the first 8 weeks (3 sessions per week) and 65% for weeks 9 to 25 [33] (2 sessions per week). These attendance rates are also consistent with what has been reported in a review of numerous randomized controlled trials of physical activity and older adults [34]. The expectation for participants to re-book missed cognitive training sessions may have motivated the participants in the combined group to attend both cognitive training and physical activity sessions. Furthermore, the PACT condition may have offered a more novel and interesting intervention that served to enhance interest and commitment and thus higher levels of attendance. Additionally, the fact that cognitive training and physical activity sessions were offered on the same day may have facilitated attendance at both sessions.

In terms of adhering to the physical activity goals, participants did not achieve the 150 minutes per week of aerobic physical activity goal. This goal was chosen because it represents the level of physical activity that is recommended by the American College of Sports Medicine [35]. However, although reporting of physical activity adherence in older adults is inconsistent across studies [34], previous research indicates that achieving recommended levels of physical activity in sedentary samples of older adults is quite difficult. In a sample of formerly sedentary older adults, the LIFE-P intervention included a phased contact schedule (3-days/week during the first 6-months) and a behavioral group-counseling module and reported approximately 128 min/wk of physical activity during the first 6 months of the trial [36]. The present study included only 4 months of physical activity, did not include a behavioral group-counseling module, and had fewer center-based contacts than LIFE-P. Future randomized clinical trials investigating the impact of physical activity on cognition should include structured behavioral modules to address the multitude of barriers to physical activity encountered by sedentary older adults to maximize adherence to study protocols [36].

### Choice of a control condition

We adopted, as our control condition, the Healthy Aging Education program of the Lifestyle Interventions and Independence for Elders Pilot (LIFE-P) trial, which had been developed to foster retention in a clinical trial of a physical activity training intervention while not directly affecting its outcomes, markers of mobility and physical function [22,23]. We found, across four months of follow-up, retention in this comparison condition to be equivalent to those of the training-based interventions, which parallels the experience of the LIFE-P trial.

A substudy, involving 102 participants, added measures of cognitive function to LIFE-P Pilot at baseline and one-year post-randomization to compare a physical activity intervention to the same control condition we used [8]. Similar to our study, no significant relative benefits were found for the physical activity intervention. Also similar, modest improvements in cognitive function were seen in the performance of participants assigned to the Healthy Aging Education program on several measures of cognitive function. These reached statistical significance for a test of verbal learning. While these may reflect learning effects, it is also possible that the Healthy Aging Education program may have affected cognitive function. Increased social engagement and reading, both features of the Healthy Aging Education program, may produce cognitive benefits in older individuals [37]. Furthermore, within the full LIFE-P trial, the Healthy Aging Education program was associated with modest mean improvements in a battery of physical function tests, which although less than for its physical activity intervention, may signal that the control condition was not benign with respect to other markers of health [23].

Choice of a control condition in behavioral interventions can be controversial from design and ethical perspectives [38,39], and active control conditions may reduce estimated effect sizes [40]. SHARP-P incorporated the control condition to enhance retention and reduce differences in exposure to study staff among intervention groups, with the aim of providing an appropriate contrast to the mechanisms specific to the physical activity and cognitive training interventions. Even so, exposure times varied among intervention conditions: these are often difficult to balance with behavioral interventions and may contribute to relative intervention effects. Although the SHARP-P control condition may produced some improvements in performance on cognitive tests, one may argue that for behavioral interventions to be adopted as part of clinical practice, they should be demonstrated to provide benefits above whatever effects the more modest and less resource intensive Healthy Aging Education program provided. However, others argue that true placebo controlled trials are necessary in situations where no effective therapies have been established and mechanisms may be non-specific [41,42].

### Short term effects of cognitive and physical training on cognitive performance

Assignment to our cognitive and physical activity interventions did not produce significant relative improvements in our overall composite measure or in the separate components focused on executive function and episodic memory after four months. As designed, the SHARP-P trial targeted the detection of intervention mean relative effects of 20% differences on domain specific tests in marginal comparisons, which was equivalent to an average of 0.64 standard deviations across domains. Thus, the observation of fairly substantial differences was required for statistical significance in this pilot study. For the composite primary outcome, post hoc power calculations from the observed standard errors of differences indicate 80% power was available to detect mean marginal differences of 0.49 standard deviations. Although SHARP-P, as conducted, provided sufficient power to detect the targeted differences, observed differences were smaller than these. Our composite outcome was projected to provide greater statistical power than its individual components if intervention effects were distributed among its components. Composite outcomes allow one to deal efficiently with problems of multiple comparisons [43]. However, if intervention effects vary markedly among individual components, some important differences may not be detected. Our results should be interpreted later in the context of larger trials.

Of great interest, functional magnetic resonance imaging was conducted at the conclusion of the trial on five participants who had been assigned to the Healthy Aging Education condition and six who had been assigned to the PA. Despite the small sample size, the hippocampi of PA participants had significantly greater cerebral blood flows and network connectivity than Healthy Aging Education participants [44]. Thus, despite the lack of an effect on our cognitive measures, four months of physical activity training may have produced beneficial effects on other measures of brain function.

Several recent small trials report stronger intervention effects on cognitive measures. Fabre, et al. found that logical memory, paired associates learning scores and memory quotient were significantly improved after two months of aerobic and/or mental training compared to a control group [5]. Smith, et al. found four months of a multi-component behavioral intervention that included physical exercise, weight loss, and diet was associated with a relative improvement in measures of executive function in overweight/obese individuals with mean age 52 years [15]. Baker, et al. found six months of aerobic exercise training produced significant benefits in measures of executive function in women; these were not evident in men and did not occur after three months of intervention [11]. Liu-Ambrose found significant improvements in executive function after 12 months of resistance training in women with mean age 70 years, but not at 6 months [10]. Albinet, et al. found 12 weeks of aerobic training improved executive function in older sedentary adults compared to stretching [9]. Three other recent pilot trials, however, found no significant intervention effects on cognitive outcomes. Stuss, et al. found three months of a memory, goal management, and psychosocial training did not significantly affect measures of memory [45]. As noted above, Williamson, et al. found no significant differences in executive function and other domains after 12 months of a physical activity intervention compared to the healthy aging control condition adopted by SHARP-P; however, an analysis pooling data across intervention groups found a significant positive correlation between improvements in physical function and cognitive function [8]. Dechamps, et al. found little improvement in global cognitive function after 12 months of an exercise program [12].

Overall, it appears that the greatest effects may be for interventions that 1) last longer than four months, 2) target executive function, 3) feature a minimal control condition and, possibly, 4) are multi-factorial. The choice of executive function as a primary outcome is supported by meta-analysis [46]. However, combining individual measures into a composite outcome, as we did, can sometimes dilute focused effects [43]. In contrast, examining neuroimaging data following intervention may provide a more sensitive outcome measure than cognitive tasks.

### Consistency across age ranges

We investigated if follow-up and intervention effects were attenuated by age in our participants. We found no evidence, across the relatively short time frame of four months, of poorer adherence rates among our older volunteers. Given issues of comorbidities and other threats to intervention participation, one might expect longer trials to face greater issues. Interestingly, however, the Diabetes Prevention Program found that older participants (>65 years of age) were more likely to achieve the physical activity and weight loss goals of the study [47]. The eligibility and recruitment approaches used in SHARP-P appeared to be effective for identifying older individuals who can be adequately adherent to the interventions being offered, at least in the short-term.

Of great potential interest is that we found a trend that physical activity training may convey greater relative short-term benefits on cognitive function among our older volunteers. Assignment to the physical activity intervention appeared to lead to improvements in cognitive function that increased, in a graded fashion, across the age range of 70-85 years. While this comparison was pre-specified, we cannot claim statistical significance due to the many inferences we conducted in our pilot study. All SHARP-P participants self-identified as having cognitive deficits; while this is known to be a weak risk factor for future cognitive decline [48,49], it is possible that this relationship may signal differences in responses between individuals who experience relatively earlier versus later declines. There is precedence for greater benefits of physical activity training among individuals with lower baseline functioning in other outcomes. For example, Marsh, et al. found baseline lower extremity functioning moderated the influence of two different walking programs on improvements in physical functioning [50]. Whereas those with higher levels of functioning responded more positively to a traditional walking program, the lower functioning participants responded more positively to a novel walking program that included more complex walking tasks, such as stepping over obstacles.

### Sample size projections for the full-scale trial

Using benchmark data generated by SHARP-P, we projected that a two-armed four-year trial to detect relative differences in cognitive deficits can be mounted with fewer than 1,000 participants (continuous outcome) or 2,000 participants (categorical outcome). The required sample sizes are dependent on event rates, and thus on the risk profiles of participants. Trials aimed at affecting the endpoints of mild cognitive impairment or dementia typically would be expected to require larger sample sizes because events rates may be lower [51].

### Limitations

While large enough to meet its objectives, our pilot trial involved a modest sample size and short follow-up. Our participants were predominantly Caucasian and reported relatively high levels of education. We examined only two training-based interventions; it is likely that the most effective programs may include tool-box approaches with multiple options [1,2]. Similarly, the physical activity program was a relatively short, traditional walking program and did not include anaerobic forms of activity, such as strength training. Evidence suggests that longer physical activity programs (>6 months) that include both aerobic and anaerobic exercise have greater effects than aerobic exercise alone [46]. Additionally, the nature of the Healthy Aging Education control condition may have influenced outcomes.

## Conclusions

Pilot studies should be designed with objectives that are attainable and with the aim of informing larger studies that are feasible and important. They are not mounted as short-term underpowered prototypes of large studies. Investigators conducting pilot studies bear the ethical responsibility of disseminating findings and, as justified by the results, holding a commitment for developing the subsequent full scale research programs.

Our results support the design of rigorous large-scale trials to assess whether training-based interventions have a role in maintaining brain health in older adults. Adequate participation, adherence, and retention appear to be achievable for participants through age 85 years, at least in the short-term. An attention control condition may yield levels of retention that are comparable to active interventions; however, care should be taken to ensure that this type of active control does not unduly attenuate intervention effects. Depending on the choice of outcome measures, the necessary sample sizes to conduct four-year trials appear to be feasible, particularly as part of a multi-site trial.

## Competing interests

The authors declare that they have no competing interests.

## Authors' contributions

CL, JMJ, JAK, DD, KMS, SRR, WJR, SAS and MAE conceived of the study, participated in its design, coordination and recruitment, and drafted and revised the manuscript. CL, JMJ, JAK, DD, SAG, KMS, SRR, and MAE were involved in training, coordination and recruitment. CL, MAE and SAG performed statistical analyses. All authors read and approved the manuscript.

## Pre-publication history

The pre-publication history for this paper can be accessed here:

http://www.biomedcentral.com/1471-2318/11/27/prepub
